# VHL syndrome without clear family history: A rare case report and literature review of Chinese patients

**DOI:** 10.3389/fneur.2022.951054

**Published:** 2022-10-17

**Authors:** Yaheng Li, Xiaohong Xin, Wenzhu Song, Xuan Zhang, Shengli Chen, Qian Wang, Aizhong Li, Yafeng Li

**Affiliations:** ^1^Department of Nephrology, Shanxi Provincial People's Hospital (Fifth Hospital) of Shanxi Medical University, Taiyuan, China; ^2^Shanxi Provincial Key Laboratory of Kidney Disease, Taiyuan, China; ^3^Core Laboratory, Shanxi Provincial People's Hospital (Fifth Hospital) of Shanxi University, Taiyuan, China; ^4^Academy of Microbial Ecology, Shanxi Medical University, Taiyuan, China; ^5^School of Public Health, Shanxi Medical University, Taiyuan, China; ^6^Department of Neurosurgery, Shanxi Provincial People's Hospital (Fifth Hospital) of Shanxi Medical University, Taiyuan, China

**Keywords:** Von Hippel–Lindaut syndrome, whole exome sequencing, gene mutation, literature, genetic testing

## Abstract

**Objective:**

To analyze the clinical manifestations and imaging features of a hospitalized patient with intermittent headache who was finally diagnosed with von Hippel–Lindau (VHL) syndrome and to perform whole-exon gene detection to improve the understanding of the diagnosis and treatment strategies of the disease.

**Methods:**

A case of suspected VHL syndrome in Shanxi Provincial People's Hospital was analyzed. Proband DNA was also extracted for whole exome sequencing and screened for causative mutation sites, which were validated by Sanger sequencing. The literature about *VHL* gene mutations in Chinese patients in the past 10 years were also reviewed.

**Results:**

There is a heterozygous mutation site c.499C > G on the *VHL* gene on the short arm of chromosome 3 of the patient, which is a missense mutation. The mutation results in the substitution of arginine with glycine at amino acid 167 of the encoded protein, which may be primarily responsible for the disease in the patient with VHL syndrome. However, the mutation did not occur in other family members.

**Conclusion:**

Early recognition and treatment of VHL syndrome can be available with genetic testing technology. Strengthening the understanding of this complex genetic disease and improving the diagnostic rate of VHL syndrome are helpful for the precise treatment of patients with this disease, which may help prolong the survival time of patients to a certain extent and improve their quality of life.

## Introduction

Von Hippel–Lindau (VHL) syndrome is a rare clinical familial autosomal dominant tumor syndrome involving multiple body systems. Characteristic tumors in VHL syndrome include central nervous system hemangioblastomas (CNS HBs), renal cell carcinomas (RCCs), pheochromocytomas (PHEOs), and pancreatic cystadenomas. The most common tumors are retinal or CNS HBs (60–80%) and VHL syndrome-related renal lesions, which may range from simple cysts to multiple and renal cell carcinomas (RCC) (24–45%)(1). VHL syndrome-associated tumors frequently lose the function of the wild-type VHL allele in the process, which is known as loss of heterozygosity (LOH). According to statistics, about 1/36,000–53,000 people have *VHL* gene mutations (2). The majority of VHL syndrome affected have a positive family history, whereas sporadic cases of VHL syndrome with possible *de novo* mutations are rare (about 20%) and may occur during germ cell formation or early embryogenesis (3, 4). The patient with a mild clinical phenotype may be considered a sporadic case in this setting. Now, we present the genomic findings in a 50-year-old man with multiple CNS HBs, RCCs, and pancreatic neuroendocrine tumors (PNETs) diagnosed as suspected VHL syndrome without a clear positive family history. It was finally confirmed that the patient with VHL syndrome had a mutation site on the pathogenic gene *VHL*, which may be the cause of the disease.

## Case presentation

### Primary concerns and symptoms

A 50-year-old man presented with a 3-month history of frequent headaches associated with vomiting, nausea, and dizziness was admitted to the hospital for intermittent headache for more than half a month, aggravated with dizziness, nausea, and vomiting for 3 days. The patient had normal diet and sleep, normal urine and defecation, no significant weight loss, and no other discomforts.

### Physical examination

The patient was generally in good condition. The systemic superficial lymph nodes were not palpable and enlarged, and the cardiopulmonary abdominal examination showed no abnormality. The patient had bilateral pupils of equal size and round, 2.5 cm in diameter, and sensitivity to light, and fundus examination showed no abnormality. Auxiliary examination: blood routine and urine routine were normal, fecal occult blood (-); serum amylase and urine amylase were normal; liver and kidney function, tumor markers, and coagulation showed no significant abnormality.

### Laboratory tests

**Figure 1:**
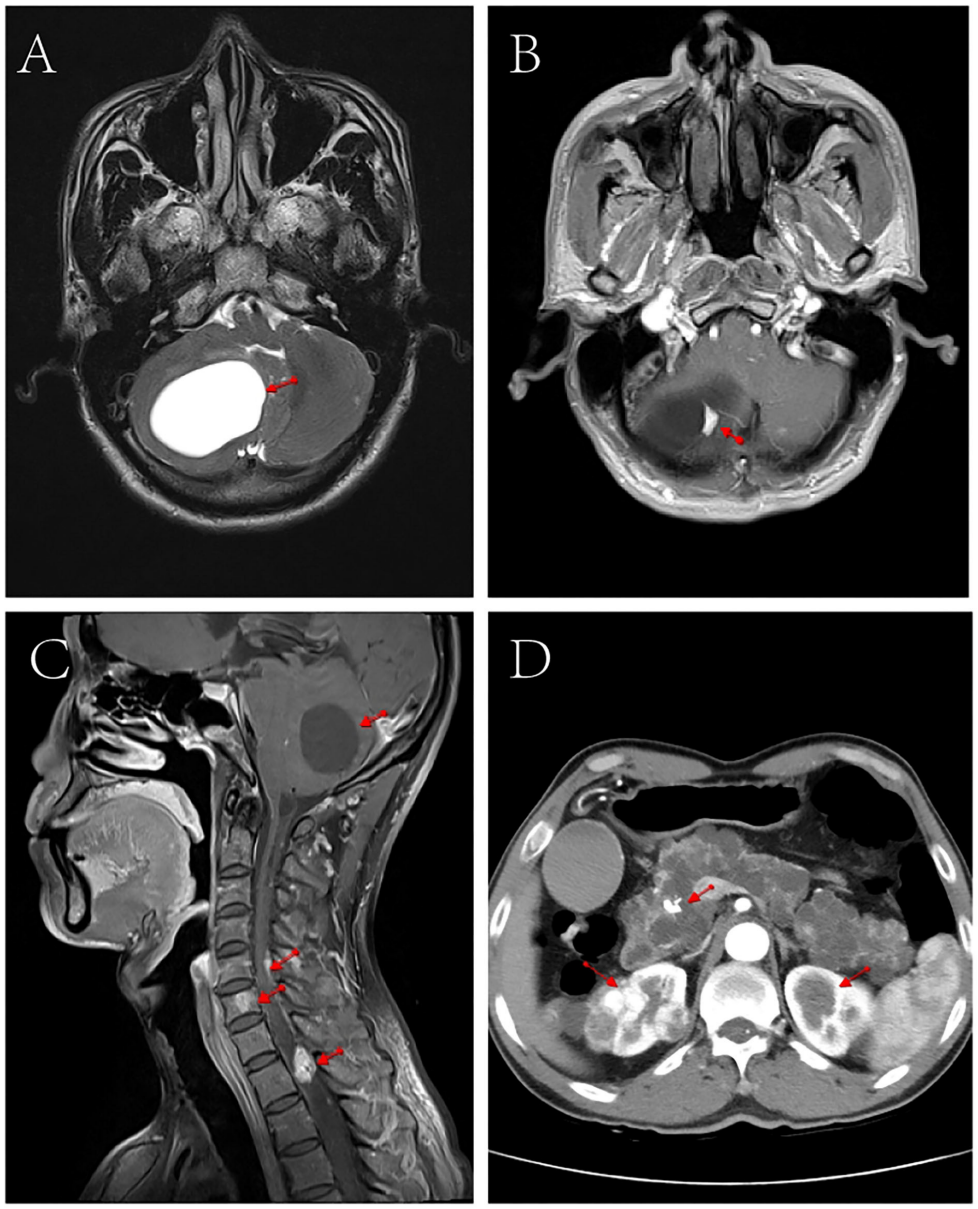
CT and MRI scan images of the patient. **(A)** Cranial MRI showed space- occupying lesions in the right cerebellar hemisphere. **(B)** Cranial enhanced MRI showed nodular enhancement in the right cerebellar hemisphere lesion (1.0 cm × 0.6 cm). **(C)** Contrast-enhanced MRI of the head and neck showed narrowing of the space in the fourth ventricle, multiple nodular and tortuous line-like enhancement in the spinal cord from C5 to T2, and round abnormal enhancement in the C6 vertebrae. **(D)** Abdominal enhanced CT showed multiple round soft tissue density lesions and multiple cystic low-density lesions in both kidneys, multiple low-density lesions of different sizes in the pancreas, and calcifications in the head of the pancreas.

Contrast-enhanced MRI of the head and cervicothoracic spine (Figure 1) showed nodular enhancement in the lesions of the right cerebellar hemisphere, about 1.0 × 0.6 cm, with clear contour; the fourth ventricle crushed and narrowed, enlargement of the lateral and third ventricles; multiple nodular and tortuous linear enhancement lesions at C5-T2 of the spinal cord, with blurred contour; and round abnormal enhancement shadows of the C6 vertebral body, with blurred contour, which presented suspected radiological diagnosis of HB. Contrast-enhanced abdominal CT scan showed multiple low-density lesions of different sizes in the pancreas and partially fused; pancreatic duct dilatation; calcification of the pancreatic head; and after enhancement, a significant round enhanced nodule of about 1.1 cm in diameter was observed on the pancreatic head, which was considered a neuroendocrine tumor. Multiple round soft tissue density lesions were observed in both kidneys, the larger one was about 3.7 cm in diameter, and the enhancement in the arterial phase was higher than that in the adjacent renal cortex and renal cancer was considered. Multiple low-density lesions were observed in both kidneys, some of which were accompanied by septum and calcification, and multiple cysts were considered.

### Operation and postoperative course

The patient underwent a large solid cerebellar cystic mass resection. Pathological diagnosis confirmed the lesion as HB.

After 2 years of follow-up, the condition of the patient was relieved and relatively stable.

### Gene analysis

To detect the *VHL* gene mutation in the patient and his family, we collected peripheral blood from the patient, his daughter, his son, and his only younger brother. Exons of *VHL* gene were amplified from genomic DNA by polymerase chain reaction. Primer pairs are shown in Table 1.

**Table 1:** Primer sequences of exons of *VHL* gene.

**Exons**	**Primer direction**	**Primer sequences**
Exon 1	Forward	5′-GCGAAGACTACGGAGGTC-3′
	Reverse	5′-ATGTGTCCTGCCTCAAGG-3′
Exon 2	Forward	5′-CCTAGACCTCATGATCCGC-3′
	Reverse	5′-TTGGATAACGTGCCTGACATC-3′
Exon 3	Forward	5′-GGTAGTTGTTGGCAAAGCCTC-3′
	Reverse	5′-GAAACTAAGGAAGGAACCAGTCC-3′

**Figure 2:**
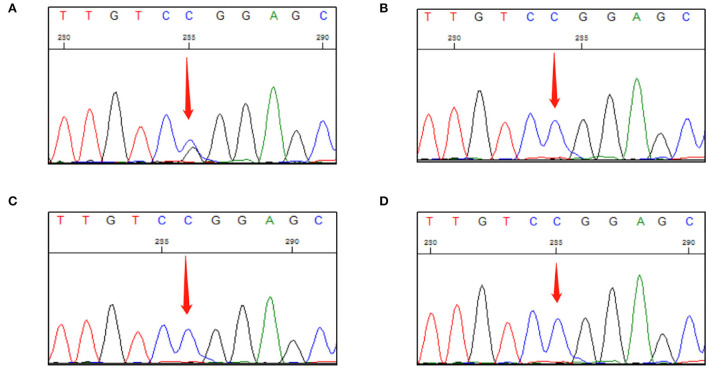
First-generation sequencing peak diagram. **(A)** The proband carried a heterozygous variant at the VHL c.499C>G site. **(B)** The proband's daughter did not carry this heterozygous mutation. **(C)** The proband's son did not carry this heterozygous mutation. **(D)** The proband's only younger brother did not carry this heterozygous mutation.

Sanger DNA sequencing was performed and each exon was identified and confirmed by forward and reverse analysis. A heterozygous mutation (c.499 C > G) in exon 3 of *VHL* gene was detected in genomic DNA from a peripheral blood sample collected from the patient. This mutation was not detected in the peripheral blood of any other family member (Figure 2). After investigating the family history of the patient, it was found that his parents, grandparents, and maternal grandparents had died. The proband's father died of chronic heart disease, and the mother was healthy before her death; however, she died unexpectedly due to a car accident. None of them had a clear history of VHL syndrome-related disease.

## Discussion

Von Hippel–Lindau syndrome is a progressive multisystem familial tumor syndrome characterized by phenotypically similar vascular tumors in the CNS and viscera. It's usually caused by germline mutations in the VHL tumor suppressor gene located on the short arm of chromosome 3 (3p25–26) (3).

At present, gene mutation types such as missense mutations, frameshift mutations, and in-frame deletions/insertional mutations have been detected in VHL syndrome patients. Depending on the presence or absence of PHEO, it can be divided into two clinical types. Patients with VHL type I have a low risk of developing PHEO (5). Most cases of VHL type I might be caused by missense mutations affecting the hydrophobic core of the protein, and the mutations lead to protein truncation, and partial gene deletions, which may be associated with VHL instability and high HIF activity, resulting in complete defects in protein function. In contrast, while type II is often subdivided into type IIA (with low risk of clear cell RCC (ccRCC)), type IIB (with increased risk of ccRCC), and IIC (pheochromocytoma only).VHL type II is often associated with missense mutations affecting the binding site of VHL protein (6).

Patients with VHL disease are subjected to an increased risk of developing HBs, which may affect 60–80% of patients (7). These tumors are usually multiple and no more than 50% of HBs will enlarge when followed up for more than 5 years of follow-up (8). The most common HBs are cerebellar or retinal capillary HBs, which can also occur in the spinal cord or brainstem (1). Although the tumor is benign, HB may also greatly increase morbidity and mortality in patients with VHL syndrome due to its significant impact on central nervous system architecture, and retinal edema, hemorrhage, detachment, and visual loss due to tumor exudation (9). The results of enhanced MRI examination of the cranial and cervicothoracic spine as well as CT examination showed that HB was present in the cerebellar of our reported case and cerebellar mass resection was performed. A fundus examination of the patient was not performed at that time because the eye examination showed normal vision, and the fundus examination showed no abnormality at the reexamination of the patient 1 year later. Since retinal capillary HB is the most common manifestation of VHL disease, the patient should be advised to examine the fundus at each reexamination to prevent the progression of the disease and delay in treatment. Multiple renal cysts were frequently found to coexist with RCC in 60% of patients with VHL syndrome (10), and CT diagnostic results showed renal carcinoma with multiple cysts, as described in our report. The frequency of RCC increases with age, with up to 70% of VHL patients developing RCC at age 60 and ultimately leading to death (11). About 10–20% of patients with VHL develop PHEO which can cause systemic paragangliomas. It has been associated with secondary hypertension or stroke and ultimately with death in 5% of patients with VHL syndrome (12). Approximately 35–70% of VHL syndrome patients have pancreatic manifestations (13), and as in this patient, CT examination showed multiple cysts of different sizes in the pancreas, with calcifications in the pancreatic head, which are considered neuroendocrine tumors. Although pancreatic neuroendocrine tumors are rarely responsible for morbidity and mortality, tumor transformation to malignancy or metastasis may result in a poor prognosis.

VHL syndrome can be clinically diagnosed in patients with positive family history and with central nervous system HB, RCC, or PHEO. Those without a family history must have two or more HBs or one HB and one visceral tumor (RCC, PHEO, or pancreatic tumor). In this case report, a 50-year-old patient was found to have multiple CNS HBs, multiple pancreatic and renal cysts, and RCC. Although the patient we reported had no clear family history of VHL syndrome, he still met the diagnostic criteria for VHL syndrome.

The c.499 C > G mutation of our reported case is expected to result in a mutation of amino acid 167 of the encoded protein from arginine to glycine. The arginine residue is highly conserved, with a moderate physicochemical difference between arginine and glycine. Algorithms developed to predict the impact of missense changes on protein structure and function (SIFT, PolyPhen-2, Align-GVGD) suggest that this variant may be disruptive, but these predictions require further confirmation by functional studies. Most patients with VHL syndrome inherit a germline mutation from one copy of the *VHL* gene in affected parents and one normally functioning wild-type allele from unaffected parents. Two studies have reported that the c.499 C > G mutation in *VHL* gene was associated with VHL syndrome (14, 15). However, one patient had sporadic PHEO, and the other patient had a positive family history of VHL syndrome, but there was no PHEO and no clear family history of VHL syndrome in this patient. Due to the death of his parents and the absence of VHL syndrome-related conditions, first-generation sequencing verification showed that his asymptomatic younger brother, daughter, and son did not carry the heterozygous mutation. The heterozygous mutation may be *de novo*. In addition, we searched the relevant literature in PubMed, Web of Science, Embase, and other literature databases for the past 10 years (January 2012–August 2022) by using keywords such as “VHL syndrome”, “VHL disease”, “gene mutation”, and “case report”, and selected the literature with more complete clinical data for summary, and 26 cases met the conditions. We summarized the *VHL* gene mutation reports of the Chinese population in Table 2. Of the 26 Chinese patients we summarized, 16 cases belonged to VHL type I and 10 cases belonged to VHL type II, and the gene mutation types involved missense mutations, frameshift mutations, deletions and duplications of bases, and alternative splicing of introns. These mutations often occurred in the second half of exon 1 (14 cases), the first half of exon 2 (3 cases), and exon 3 (6 cases). The study has shown that *VHL* gene mutations often occurred at codons 75 and 82, cleavage sites between exon 2 and exon 3, and codons 157–189, and the hotspot of mutations was at codon 167 (CpG island region) (35). In addition to the common VHL syndrome-related neoplasms, our summary also showed that nonobstructive azoospermia, clear cell peritoneal epithelioid mesothelioma, and familial polycythemia type 2 were also associated with VHL syndrome, and the basic main treatment modalities were surgical resections or chemoradiotherapy. They are mainly based on point mutation and splice mutation. By detecting the characteristics of gene mutations and comprehensively considering family characteristics, clinical manifestations, and pathological diagnosis, it is helpful to deeply study the pathogenesis and pathogenic characteristics of VHL syndrome, thereby enriching the knowledge of this disease.

**Table 2:** *VHL* gene mutations in Chinese patients.

***VHL*** **mutations**	**Locations**	**Consequences**	**Clinical features**	**Therapies**	**VHL type**	**References**
c.193 T > C	Exon 1	p.Ser65Pro	CNS HBs, multiple RCC, and pancreatic cysts	Surgical resections	VHL I	(16)
c.194 C > G	Exon 1	p.Ser65Trp	Multiple RCC and pancreatic cysts	Surgical resections	VHL I	(16)
c.208 G > A	Exon 1	p.Glu70Lys	CNS HBs, RCC, colorectal adenocarcinomas.	Surgical resection or radiation therapy	VHL I	(17)
c.227_229 del TCT	Exon 1	p.Phe76del	CNS HBs, solid kidney tumor	Surgical resection for HBs	VHL I	(18)
c.232 A > T	Exon 1	p.Asn78Tyr	Right RCC	Radical nephrectomy and molecular targeted drug Sunitinib	VHL I	(19)
c.233 A > G	Exon 1	p.Asn78Ser	Right PHEO and right RCC, bilateral epididymal cyst	Right PHEO and RCC resection and bilateral epididymal cyst resection	VHL IIB	(20)
c.239 G > T	Exon 1	p.Ser80Ile	RCC, HBs, PHEO	Surgical resection for HBs and PHEO	VHL IIB	(19)
c.254 dup T	Exon 1	p. Leu85fs	Peritoneal epithelioid mesothelioma of clear cell type	Cytoreductive surgery (CRS) and hyperthermic intraperitoneal chemotherapy (HIPEC) operation	VHL I	(21)
c.256 C> T	Exon 1	p.Pro86Ser	CNS HBs, RCC, PHEOs, multiple cysts in the pancreas and liver	Tumor resections	VHL IIB	(22)
c. 257 C > T	Exon 1	p.Pro86Leu	Optic nerve HB, pancreatic cysts	Surgical resection for HB	VHL I	(23)
c.262 T > C	Exon 1	p.Try88Arg	RCC, nonobstructive azoospermia, bilateral renal cysts, and pancreatic cysts	Not described	VHL I	(24)
c.264 G > A	Exon 1	p.Trp88Trp	PHEOs, RCC	Surgical resections	VHL IIB	(25)
c.293 A > G	Exon 1	p.Try98Cys	PHEOs	Not described	VHL IIC	(19)
327 del C	Exon 1	p.Gly39Alafs*26	CNS HBs	Surgical resection for HBs	VHL I	(26)
c.340+5 G > C	Intron 1	Abnormal splicing	Cerebral HB, PHEOs, liver cysts	Surgical resections	VHL IIA	(22)
c.349 T > A	Exon 2	p.Trp117Arg	Cerebellar and retinal HBs	Surgical resection for HBs	VHL I	(27)
c.386 T >G	Exon 2	p.Leu129Pro	PHEO, PNET	Tumor resections	VHL IIA	(28)
c.451 A > T	Exon 2	p.Ile151Phe	Cyst-solitary papilloma, pancreatic cysts, RCC, Polycystic kidney, cystadenoma in the bilateral adnexal nodes	Multiple tumor resections, Clarithromycin	VHL I	(29)
c.464T > A	Exon 3	p.Val155Glu	CNS HBs, PHEOs	Surgical resection for HBs	VHL I	(30)
c.464-1G > C	Intron 2	Abnormal splicing	CNS HBs, multiple cysts in the pancreas and kidneys	surgical resection for cerebellar HBs	VHL I	(31)
c.464-2A > G	Intron 2	Abnormal splicing	Multiple HBs, RCC, and multiple renal and pancreatic cysts	surgical resection for HBs in the bilateral cerebellum	VHL I	(31)
c.482 G > A	Exon 3	p.Arg161Gln	Right PHEO	Right PHEO resection	VHL-IIB	(20)
c.499 C > T	Exon 3	p.Arg167Trp	Bilateral renal tumor, bilateral renal cyst CNS HBs, RCC, PHEO	Surgical resections	VHL IIB	(32)
c.500G > A	Exon 3	p.Arg167Gln	PHEO and left renal cyst	Laparoscopic surgery	VHL-II	(19)
c530 536delGACTGGA	Exon 3	P.Arg177fs	HB in the cerebellum, bilateral polycystic kidney disease, multiple hepatic cysts, and pulmonary nodules	Ventriculoperitoneal shunt and postoperative radiotherapy	VHL-I	(33)
c.598C > T	Exon 3	p.Arg200Trp	Familial erythrocytosis type 2	Not described	VHL I	(34)

The protein pVHL encoded by the *VHL* gene is a master regulator of hypoxia-inducible factor-α (HIF-α). pVHL consists mainly of α domain and β domain. The α domain mainly forms a VCB-CUL2 complex with elongin B and elongin C, further constituting E3 ubiquitinase (36). The β domain mainly interacts with the hypoxia-inducible factor HIF-α (37). Under normoxic conditions, the VCB-CUL2 complex directly acts on HIF-α for polyubiquitination and degradation. Mutations in the *VHL* gene may reduce the degradation ability of E3 ubiquitinase, resulting in the accumulation of HIF-1α protein, which in turn activates key carcinogenic pathways such as angiogenesis, glycolysis, glucose transport, and erythropoiesis (38, 39). The upregulated cytokines such as vascular endothelial growth factor (VEGF), platelet-derived growth factor (PDGF), erythropoietin, and transforming growth factor alpha (TGF-α) (40, 41) may lead to abnormal proliferation of tumor cells, inhibit cell apoptosis, and promote the tumor occurrence. The c.499 C > G mutation we reported is located in the α domain and may affect the binding of VHL to elongin C and affect a series of downstream biological responses (42, 43), leading to the onset and progression of disease.

The treatment of VHL syndrome is mainly based on the surgical treatment of lesions in various organ systems to remove the lesion and relieve symptoms, but it cannot fundamentally improve the prognosis. With precision medicine and sequencing technology springing up, the diagnosis and treatment of VHL syndrome have gradually opened a new chapter. The use of drugs that block the pro-angiogenesis of VEGF signal transduction pathway can block the occurrence and development of the disease to a certain extent. Studies have shown that the use of anti-VEGF drug bevacizumab in patients with metastatic renal cancer can improve the survival rate of patients to some extent (44). However, bevacizumab may have a moderate impact on retinal HBs (45, 46). The use of the receptor tyrosine kinase inhibitor sunitinib can reduce the volume of PHEOs as well as renal and pancreatic tumors (47). In 2018, Zhang et al. discovered a new *VHL* target ZHX2 (zinc fingers and homeoboxes 2). ZHX2 may regulate nuclear factor B (NF-KB) signaling in VHL-deficient tissues to promote RCC tumorigenesis, potentially becoming a new target for the treatment of RCC (48). In 2021, the US FDA approved HIF-2α inhibitor Belzutifan (Welireg) for the treatment of CNS HBs, RCCs, or PNET associated with VHL syndrome that does not require immediate surgery (49). With the approval of Belzutifan, the evolving technology of next-generation sequencing (NGS) may power impetus for the precision medicine of VHL syndrome-related tumors (50). With the continuous deepening of the clinical treatment of *VHL* gene and VHL syndrome, surgical treatment, targeted drug therapy, gene therapy, and other methods will enable patients with VHL syndrome to obtain a wider treatment space and more effective symptomatic treatment options.

The *VHL* gene mutation c.499 C > G in the patient we reported had no clear family history of VHL syndrome. Additionally, this patient had only a mild clinical phenotype of VHL disease. Even if there is no evidence of other VHL-related lesions or a positive family history of VHL disease, clinicians should consider the possibility of sporadic VHL in such patients. Attention should be paid to differential diagnosis and regular review to try to avoid reducing misdiagnosis and missed diagnoses. Meanwhile, molecular genetic testing of such patients and family members should be considered to help identify at-risk family members to improve diagnostic certainty and advance appropriate treatment and prevent disease progression. To improve the treatment of tumors such as CNS HBs and RCCs associated with VHL syndrome, it is necessary to continuously study new pathogenesis, discover new molecular targets, and ultimately improve the quality of life of patients.

## Data availability statement

The datasets presented in this article are not readily available because of ethical and privacy restrictions. Requests to access the datasets should be directed to the corresponding author.

## Ethics statement

The studies involving human participants were reviewed and approved by Ethics Committee of Shanxi Provincial People's Hospital. The patients/participants provided their written informed consent to participate in this study. Written informed consent was obtained from the individual(s) for the publication of any potentially identifiable images or data included in this article.

## Author contributions

YahL and XX were responsible for the drafting of the manuscript. WS helped polish the manuscript. XZ and SC offered precious knowledge on medical imaging. QW and AL were responsible for acquisition of the relevant clinical data. YafL was responsible for the conception and design of the article. All authors contributed to the manuscript and approved the submitted version.

## Conflict of interest

The authors declare that the research was conducted in the absence of any commercial or financial relationships that could be construed as a potential conflict of interest.

## Publisher's note

All claims expressed in this article are solely those of the authors and do not necessarily represent those of their affiliated organizations, or those of the publisher, the editors and the reviewers. Any product that may be evaluated in this article, or claim that may be made by its manufacturer, is not guaranteed or endorsed by the publisher.
